# From grid cells to place cells with realistic field sizes

**DOI:** 10.1371/journal.pone.0181618

**Published:** 2017-07-27

**Authors:** Torsten Neher, Amir Hossein Azizi, Sen Cheng

**Affiliations:** 1 Institute for Neural Computation, Ruhr University Bochum, Bochum, Germany; 2 International Graduate School of Neuroscience, Ruhr University Bochum, Bochum, Germany; 3 Department of Psychology, Ruhr University Bochum, Bochum, Germany; Georgia State University, UNITED STATES

## Abstract

While grid cells in the medial entorhinal cortex (MEC) of rodents have multiple, regularly arranged firing fields, place cells in the cornu ammonis (CA) regions of the hippocampus mostly have single spatial firing fields. Since there are extensive projections from MEC to the CA regions, many models have suggested that a feedforward network can transform grid cell firing into robust place cell firing. However, these models generate place fields that are consistently too small compared to those recorded in experiments. Here, we argue that it is implausible that grid cell activity alone can be transformed into place cells with robust place fields of realistic size in a feedforward network. We propose two solutions to this problem. Firstly, weakly spatially modulated cells, which are abundant throughout EC, provide input to downstream place cells along with grid cells. This simple model reproduces many place cell characteristics as well as results from lesion studies. Secondly, the recurrent connections between place cells in the CA3 network generate robust and realistic place fields. Both mechanisms could work in parallel in the hippocampal formation and this redundancy might account for the robustness of place cell responses to a range of disruptions of the hippocampal circuitry.

## Introduction

Place cells in the CA regions of the hippocampus [1] and grid cells in the medial entorhinal cortex (MEC) [2] are important components of the navigation system in mammals [3]. Place cells fire spikes selectively when the animal passes through small regions of space, which are called place fields. Whereas place cells have just one or a few place fields, grid cells fire spikes in many fields that are arranged on a hexagonal grid.

Both cell types are similarly dependent on landmarks and boundaries of the environment. They exhibit stable firing patterns during repeated visits to the same environment [4], are robust to the removal of some environmental cues [2, 5], mostly preserve their firing maps in darkness [6, 7], rotate their spatial firing maps in concert with displaced landmarks [2, 8], rescale the size of the place fields when the environment is expanded [9, 10], and remap their representations simultaneously [11, 12]. Moreover, the field sizes of both cell types increase along the dorsoventral axis [11, 13], consistent with topographic projections from EC to the hippocampus along the same axis [14].

Consequently, it has been suggested that grid cells are responsible for driving place cell activity [15–20] Theoretical models have shown that it is indeed possible to create place cells from grid cells in a simple feedforward network by competitive learning [17, 21], through competitive activation [22], by Fourier transformation [18], by assigning weights in a specific manner [23], by Hebbian learning [24], by independent component analysis [20] or by applying linear regression [19].

However, recent experimental findings call into question the plausibility of such a simple relationship. Stable place cells were found in the hippocampus, even when the periodic firing map of grid cells were disrupted by medial septum inactivation [25, 26]. During development, mature place cells emerge before mature grid cells do [27, 28]. When two sets of cues are rotated in different directions, cells in the MEC follow global cues and place cells local cues [29, 30]. Although we showed previously that some of these issues could be accounted for by robustness of the grid-to-places transformation [31], we find here that all current models suffer from another issue that has received little attention so far: unrealistically small place field size.

Extant models produce average place field sizes ranging from about 300–627cm^2^ (Table 1) or the resulting place fields are highly sensitive to noise [23]. The average place field sizes in the robust models correspond roughly to the small place fields of granule cells in the rat dentate gyrus [33]. However, in the CA regions, place fields are significantly larger. Place cells in the dorsal CA regions have fields size of around 1225cm^2^ in CA3 and 1775cm^2^ in CA1 [34]. Moreover, place fields as large as 5000cm^2^ have been reported for dorsal cells in both regions.

**Table 1:** Comparison of place field sizes and numbers in selected studies.

Study	Field Size	Number	Reference
Model (competitive learning)	350cm^2^	1.2	[21]
Model (competitive activation)	627cm^2^	1.5	[22]
Model (random weights; CA3)	290cm^2^	1.1	[32]
Model (predefined weights)	< 420cm^2^	1	[31]
Measurement DG	< 120cm^2^	1-4	[33]
Measurement dorsal CA3	1275cm^2^	1.5	[34]
Measurement dorsal CA1	1725cm^2^	1.4	[34]

Here we first use a general feedforward model to show that the problem arises from the structure of the spatial autocorrelation of grid cells and hence cannot be avoided by tuning parameters in the specific models. We then propose two alternative models that can produce realistic place fields. First, a feedforward network that receives inputs from grid cells and weakly spatially modulated cells, which appear to be abundant in the entorhinal cortex (EC). In the medial EC (MEC), there are boundary cells [35, 36], head direction cells [37], irregular spatial cells or nonspatial cells [38]. In the lateral EC (LEC), cells are receptive to individual items such as odours [39] or objects [40–43] and hence they express only little spatial specificity in object-poor environments [44, 45]. Second, a network with recurrently connected CA3 neurons that each receives narrowly tuned spatial drive (from grid cells). Since neurons recurrently excite other neurons that receive spatial inputs at a more distant location, the place field of a given CA3 cell will appear larger than the extent of the external spatial input. The two models might represent redundant mechanisms for generating place cells in the hippocampal formation. This redundancy could account for the observed robustness of place cell responses to experimental disruptions of the hippocampal circuitry.

## Materials and methods

All calculations were performed in a 2 × 1m rectangular environment, which is discretized into 80 × 40 = 3200 location bins.

### Model of inputs to place cells

#### Grid cell activity

**Fig 1:**
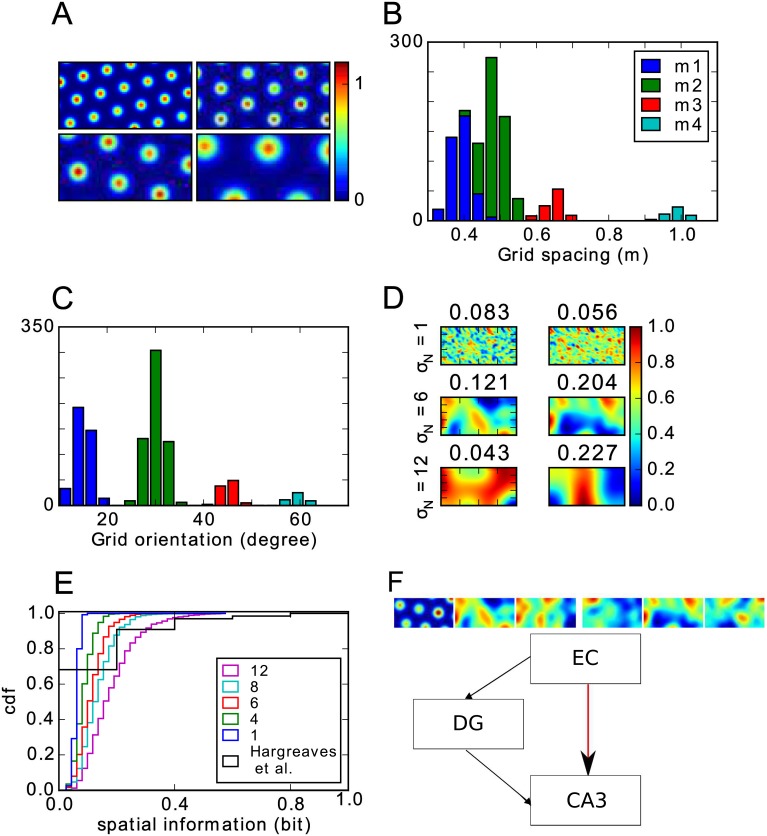
Model setup. **A**: Rate maps of four grid cell examples, one out of each module. **B-C**: Distribution of grid spacings (B) and orientations (C) in the grid cell population. Colours indicate grid module. **D**: Examples of weakly spatially modulated cells created with different kernel sizes (shown on the left). The numbers above each panel indicate the spatial information of the rate map. **E**: Distribution of spatial information for different kernel sizes. Black line shows the observed distribution in the rat LEC [44]. **F**: Overview of the modelled subregions. Black lines indicate connections that are fixed and used only during the learning of the grid to place transformation. Learning occurs in the plastic connections indicated by the red line. Only these connections drive CA3 activity once learning is complete. Rate maps illustrate the mixture of the input: 1/6 consists of grid cells and 5/6 of weakly spatially modulated cells.

The grid cell parameters in our model were chosen to match experimental data from [46] because that study recorded from the dorsal 50% of the MEC and this area probably covers all MEC inputs to a typical dorsal CA3 cell due to the topography of these projections [14, 47, 48]. The grid cell population is divided into four modules [46]. Cells in the same module have similar grid spacings and orientations, which were drawn from normal distributions (Fig 1A–1C). The grid spacings *s*_*i*_ in the four modules have a mean of 38.8, 48.4, 65 and 98.4 cm and a common standard deviation of 8 cm. Most grid cells (87%) belong to the two modules with small spacings (see Fig 1C) [46]. The orientations have means of 15, 30, 45 and 60 degrees and a standard deviation of 3 degree. The grid offset is chosen randomly from a uniform distribution.

As in previous models [24, 49, 50], the activity of each grid cell consists of multiple firing fields that are arranged in a hexagonal grid. The activation *p*_*i*_ of grid cell *i* at location **r** = (*x*, *y*) is determined by

$$\begin{array}{c} p_{i}\left(r\right)=A_{ij}\text{ exp }\left[-\text{ln}\left(5\right){\left(\frac{d\left(r\right)}{\sigma_{i}}\right)}^{2}\right], \end{array}$$ (1)

where *d* is the Euclidean distance to the nearest field center *j* and *σ*_*i*_ is the radius of the firing field. Each field has the same size, which is related to the grid spacing via *σ*_*i*_ = 0.32*s*_*i*_ (see Fig S4G in [2]). The grid cell activations are defined such that the activation reaches the peak firing rate *A*_*ij*_ in the center and 1/5*A*_*ij*_ at the border of a field, which is motivated by the definition of a place field [2]. The peak firing rates *A*_*ij*_ are drawn from a normal distribution with mean 1 and standard deviation 0.1.

#### Weakly spatially modulated cells

The rate map of an EC cell that is not a grid cell is created by assigning each location a random activation, drawn from a uniform distribution between 1 and 0. The map is then smoothed with an isotropic Gaussian kernel. The standard deviation of the smoothing kernel *σ*_*N*_ varies from 1 to 16 cm. Firing rates are then normalized such that they are between zero and one. Examples of rate maps produced by different kernel widths are shown in Fig 1D. As the default, we chose *σ*_*N*_ = 6 cm, which matches roughly the spatial information of cells in rat LEC [44, 45] (see Fig 1E). However, we analyse the effects of using other kernel widths, too. Note that we do not claim that weakly spatially modulated cells respond to the spatial location of the animal *per se*, instead we think it likely that these cells respond to other stimuli that happen to be located in a particular spatial location. For some cells, such as border cells [35], these stimuli are known, but for many other EC cells the preferred stimuli remain unknown.

### Models of place field generation

To explore how place fields with realistic place field sizes could be generated in CA3, we use three different network models. First, a general feedforward network with grid cell input only. Second, a feedforward network driven with a mixture of inputs from grid cells and weakly spatially modulated cells. Third, a recurrent network driven with inputs from grid cells.

#### General feedforward network

We adopt a generic feedforward network driven by inputs from grid cells to investigate the issue of generating realistic place fields, in principle, The network consists of an input layer containing grid cells and an output layer containing purported place cells. We denote the population vector (PV) of grid cell activity at location **r** as **p**(**r**). Each output cell *i* is activated by grid cell inputs weighted by the vector **w**_*i*_.

$$\begin{array}{c} h_{i}\left(r\right)=w_{i}^{T}p\left(r\right) \end{array}$$ (2)

Monotonic activation function *f*(*h*_*i*_) is applied to determine where the output cell fires spikes. Suppose we want cell *i* to have a large place field at location **r**_*i*_ with radius *R*_*i*_, i.e., the neuron should fire spikes when the animal is located inside the field and not elsewhere. Since the activation function *f* is monotonic, the activation *h*_*i*_(**r**) must be higher than some threshold *c* within the field and lower than the threshold outside it.

$$\begin{array}{cc} w_{i}^{T}p\left(r\right)\geq c & \forall r:||r-r_{i}\left|\right|\leq R_{i} \end{array}$$ (3)

$$\begin{array}{cc} w_{i}^{T}p\left(r\right)<c & \forall r:||r-r_{i}\left|\right|>R_{i}. \end{array}$$ (4)

Up to here, the model is general and subsumes several previous models [17–19, 21, 22, 24, 31]. Specific models differ only in the activation function, the way the weights are set up and in the choice of the threshold *c*.

The problem of finding the weight vector and threshold can be regarded as a classification problem. That is, the PVs have to be classified into two classes: PVs at locations within the place field, and PVs located outside the place field. Support vector machines are efficient algorithms for solving such problems. This classifier does not only find a solution when it exists, but also returns the solution that is most robust, in the sense that the margin to the threshold *c* is maximal (see for example [51], chapter 4.5.2]). We use the LinearSVC implementation of the python package sklearn [52] to find the weight vector and threshold for circular place fields with a radius of 10cm, 25cm and 35cm.

#### Feedforward network with inputs from grid cells and weakly modulated cells

We adopt a reduced version of the hippocampal circuit model described in detail previously in [50] to study the transformation from grid cells to place cells in a feedforward network. Briefly, our model consists of the EC, DG and CA3 (Fig 1F) with parameters that are based on the rat hippocampus. Cell numbers are 1100, 12000 and 2500 respectively. Each DG cell receives input from 352 EC cells and each CA3 cell from 7 DG cells and 352 EC cells. The sparsity, i.e., the proportion of cells simultaneously active at a given time and location, is 0.0078 in the DG and 0.032 in CA3. Note that these numbers are necessarily lower than the fraction of cells that have place fields anywhere in the environment.

In this model, the EC input to the hippocampus (DG and CA3) is provided by grid cells and weakly spatially modulated cells. To study the role of grid cells and non-grid-cells in the genesis of place cells, we perform simulations with different proportion of grid cell inputs, from 0 to 1. Unless specified otherwise, the proportion is set to the default value of 1/6, thus $1100*\frac{1}{6}=183$ cells are grid cells.

As the activation function *f* in CA3, we use a simple *k*-Winner-Take-All (WTA) mechanism: After calculating the activations of all cells in the population of place cells, the *k* cells with the highest activation are set to *h*_*i*_. The others are inhibited and set to 0. The number *k* is determined by the sparsity *a* of that region, i.e *k* = *aN*. As a result, the effect of inhibitory cells, even though not modelled explicitly, are nevertheless included in the network dynamics [49, 50, 53–57].

The weights EC-DG-CA3 are random and kept fixed (black arrows in Fig 1F), so that the DG functions as a pattern separator by mapping similar input pattern from EC onto distinct output patterns in CA3. The EC-DG-CA3 pathway serves to drive CA3 activity during learning [58], so that the synaptic weights from EC to CA3 (red arrow in Fig 1F) can be learned through Hebbian hetero-association by the Stinger rule [59]

$$\begin{array}{c} w_{ij}=\sum_{r}[p_{j}\left(r\right)-{\bar{p}}_{j}]q_{i}\left(r\right), \end{array}$$ (5)

where the summation runs over all locations **r**, *p*_*j*_(**r**) is the activity of the presynaptic cell *j*, ${\bar{p}}_{j}$ its mean, and *q*_*i*_(**r**) is the activity of the postsynaptic cell *i*. After learning, activity in CA3 is solely driven by the EC in order to determine whether the transformation from EC to CA3 is able to create place cell firing. Thus, the DG does not contribute to retrieval as suggested previously [58]. Previous studies showed that either the post-synaptically gated plasticity [24] or a k-winner-take-all mechanism [22] alone could generate place field response from grid cell inputs. However, each single mechanism requires some degree of fine tuning. For instance, the post-synaptically gated plasticity requires that the initial synaptic weights be just so, not so strong that they induce the post-synaptic cell to be active everywhere and not so weak that the post-synaptic cell is silent. In contrast, the competitive mechanism requires that synaptic weights on postsynaptic cells are randomized. Including both features in our model relaxes these constraints and makes the model more robust.

#### Recurrent model of CA3

A CA3 network that is driven by inputs from grid cells only, might generate place fields of realistic size due to its recurrent dynamics. Our recurrent CA3 model consists of 2000 excitatory (*s* = E) and 500 inhibitory (*s* = I) integrate-and-fire neurons. The dynamics of each neuron is governed by

$$\begin{array}{c} \frac{\text{d}v_{i}^{s}}{\text{d}t}=-\frac{v_{i}^{s}}{\tau_{\text{cell}}^{s}}+I_{\text{bias}}+I_{i}^{sE}-I_{i}^{sI}+I_{i}^{\text{ext}}, \end{array}$$ (6)

where $v_{i}^{s}$ is the membrane potential of the *i*-th cell, *τ*_cell_ is the integration time constant of the cell, and *I*_bias_ is a constant input current to each cell. An external input current is applied to each cell to mimic spatially selective input with a small spread

$$\begin{array}{c} I_{i}^{\text{ext}}\left(t\right)=\frac{1}{\sqrt{2\pi }\sigma_{\text{ext}}}\text{exp}[-\frac{|r_{i}{-r\left(t\right)|}^{2}}{2\sigma_{\text{ext}}^{2}}] \end{array}$$ (7)

The vector **r**_*i*_ represents the randomly assigned center of the place field of the *i*-th cell, **r**(*t*) is the location of the virtual animal at time *t* and *σ*_ext_ = 4cm is the width of the spatially selective external input. The synaptic input currents are defined as follows:

$$\begin{array}{c} \frac{\text{d}I_{i}^{ss^{\prime }}}{\text{d}t}=-\frac{I_{i}^{ss^{\prime }}}{\tau^{s^{\prime }}}+\sum_{j,k}W_{ij}^{ss^{\prime }}\delta \left(t-t_{jk}\right), \end{array}$$ (8)

where $W_{ij}^{ss^{\prime }}$ is the synaptic weight from cell *j* to cell *i*, which belong to subpopulations *s*′ and *s*, respectively, and *t*_*jk*_ is the *k*-th spike of neuron *j*. The connectivity between the populations I → E, E → I, and I → I is all-to-all. The weights are drawn from uniform distributions between a minimum value of 0 and a maximum value of 0.05, 0.1, and 0.17, respectively. Thus, the excitatory cells receive global inhibitory feedback from the population of inhibitory cells.

The connection weights between the excitatory cells are defined to produce a bump-attractor network:

$$\begin{array}{c} W_{ij}^{\text{EE}}=\frac{1}{\sqrt{2\pi }\sigma_{\text{W}}}\text{exp}[-\frac{|r_{i}-r_{j}{|}^{2}}{2\sigma_{\text{W}}^{2}}], \end{array}$$ (9)

where *σ*_W_ is the width of the connectivity kernel. The local kernel implies a topographic representational space and might be the result of a self-organizing learning process during development, much like the process suggested for the grid cell network [16]. However, this topographic representational space does not have to be reflected in the anatomical space in CA3 since the nodes in the representational space can be arbitrarily located in anatomical space. In fact, no topography has been reported in the hippocampus [60] Multiple spatial representations can be stored in the network, so that it can account for global remapping between distinct environments [61–63]. To the best of our knowledge, bump-attractor networks have not been used to explain the size of place fields previously, even though such models have been used to model place cells more generally [63] and to define the tuning width of the cells in the visual system [64].

The virtual animal explores the environment randomly for a period of 160 seconds. The average velocity of the movement is 7 cm/s. When the trajectory of the virtual animal reached the borders of the environment, the direction of the movement reversed. This particular exploration of the environment is enough to sample most regions of the environment. Longer exploration time did not change specific simulation runs (data not shown) and therefore we used the mentioned movement parameters to sample place-related activity.

### Analysis

#### Place field analysis

To calculate the place fields of the spiking neuron model, we first divided the environment into 80 × 40 bins. The firing rate map was calculated by dividing the number of spikes by the time the virtual animal spends in each bin. The firing rate map was then smoothed with a Gaussian kernel with the width of 5cm.

A contiguous region is considered a place field if a cell is active in all bins within this region and has an area > 200 cm^2^. A cell is defined as active in a spatial bin if its firing rate is at least 20% of the peak firing rate of the cell. We compare our simulation results to the data obtained by [34] who use a similar definition of a place field. Spatial information in the rate map of cell *i* is computed by

$$\begin{array}{c} I_{i}=\sum_{r}p\left(r\right)\frac{\lambda_{i}\left(r\right)}{\bar{\lambda_{i}}}\log_{2}\frac{\lambda_{i}\left(r\right)}{\bar{\lambda_{i}}}, \end{array}$$ (10)

where *p*(**r**) is the occupancy probability, which is uniform across the environment in our simulations. The value *λ*_*i*_(**r**) is the firing rate at location **r** and $\bar{\lambda_{i}}$ is the mean firing rate of the cell over all bins [65].

### Cell lesioning

To compare the robustness of the place cell spiking in our models to experimental observations after partial lesions of the entorhinal inputs, we manipulated the input in our model by A) setting the firing rate of randomly chosen input cells to zero at all locations or B) removing the place specific input current, Eq 7, to the cells. We then quantified the error rate of a downstream place cell as the average proportion of bins, in which the place cell erroneously fired or remained silent.

$$\begin{array}{c} \varepsilon =\frac{1}{2}(\frac{N(\text{silent }\&\text{ infield})}{N(\text{infield})}+\frac{N(\text{active }\&\text{ outfield})}{N(\text{outfield})}), \end{array}$$ (11)

where *N*(.) indicates the number of bins that matches the text label. The maximum error, when the cell’s firing rate is a random number, is *ε* = 0.5. This level is reached when all input cells are lesioned. On the other hand, if no noise is applied, *ε* = 0. For a network that generates a place field, but is sensitive to noise, we expect that the error rate as a function of the lesion size is a line that passes through (0, 0) and (*N*, 0.5), where *N* is the size of the network (*N* = 1100 in our case). For a place cell that is robust to noise we expect that the error rate grows slower than linear for small lesions. To more easily compare our model to experimental lesion studies, we split the EC input into MEC and LEC inputs in some simulation runs. The LEC in our model consists of 550 weakly spatially modulated cells, which may be unstable (see below). The MEC consists of 550 cells where one third are grid cells and two thirds are weakly spatially modulated cells, which are always stable.

### Stability

Since spatial rate maps of LEC cells are not as stable as those of MEC cells during a recording session or between sessions [44, 45], we tested how the instability in LEC cells might affect the stability of place cells in the hippocampus. To model instability parametrically, we first generate for each LEC cell two independent rate maps *M*_1_ and *M*_2_. The cell’s rate map on the first entry is *M*_1_. On the second entry, it is a mixture of the two maps

$$\begin{array}{c} M_{x}=\alpha M_{1}+\left(1-\alpha \right)M_{2}, \end{array}$$ (12)

where the parameter 0 ≤ *α* ≤ 1 controls for the degree of stability. The higher *α*, the higher the stability of the cell’s firing rate map across the two sessions. After applying Eq (12), we normalize the rates to ensure that they are between 0 and 1.

The weights in the feedforward network are trained on *M*_1_. We then compare the response of the hippocampal layer in this network when it is driven with either *M*_1_ or the mixed map in the LEC input, along with the identical MEC input. Like in [44], we define a cell’s stability between visits to the same environment as the correlation between the cell’s rate map on first entry and the rate map on the second entry. Furthermore, we investigate hippocampal stability when entorhinal regions are lesioned on the second entry.

## Results

### Analysis of place field size in feedforward grid-to-place transformation

**Fig 2:**
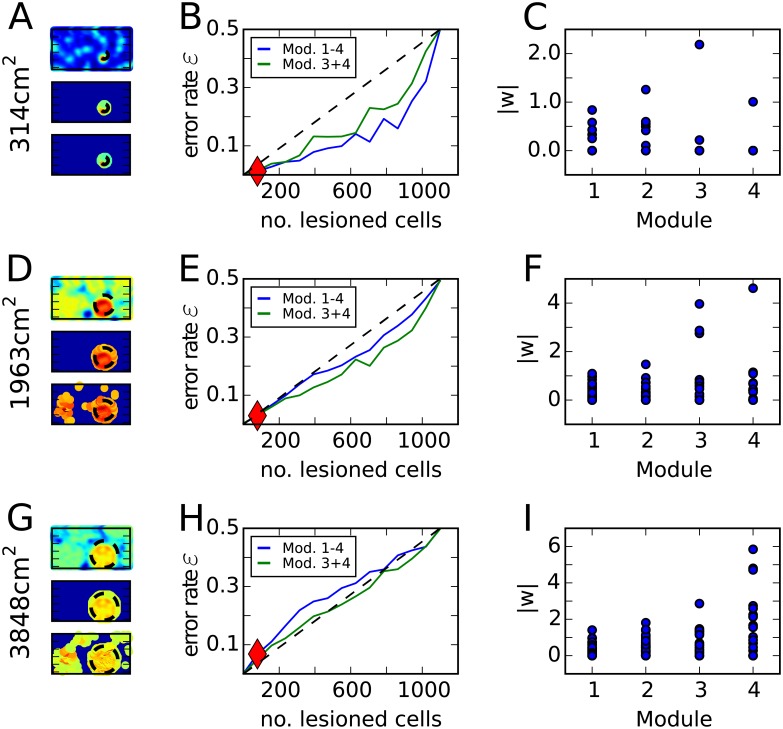
Solution of the grid-to-place transformation by a linear support vector classifier. **A**: Upper panel shows the activation map of the output cell *h*(**r**) after solving Eq 3 for a place field with a radius 10cm. Middle panel shows the map when place cells below background firing threshold have been inhibited. Lower panel shows the same as the middle panel after 7% of the grid cells have been lesioned. **B**: The error rate in the output rate map (maximal error is 0.5, see Methods) as a function of the fraction of grid cells that are lesioned is an indicator of the robustness of the solution. Blue line represents simulations when all four grid cell modules are present in the input. Green line represents simulations when only the two modules with the largest grid spacings are included. Dashed line is the reference when the error rate would increase linearly. Red diamond indicates noise level for the lower two rate maps in (A). **C**: Absolute value of the weights that are assigned to grid cells in different modules in the solution. Module one contains cells with smallest spacings and module four cells with the largest spacings. **D-I**: Same as (**A-C**) for a place field with radius 25cm (D-F) and 35cm (G-I), respectively.

To study the discrepancy in place field sizes between experiments and feedforward models, we first adopted a general two-layer feedforward network to represent the simple grid-to-place transformation (see Methods). The general problem is to find a weight vector **w** that divides the set of input population vectors (PVs) into two groups, in-field PVs and out-of-field PVs. This classification problem can be solved by a linear support vector classifier (see Methods). We train the classifier to produce place fields of different sizes: a circle with radius 10cm, 25cm or 35cm (field sizes of 314cm^2^, 1963cm^2^ and 3848cm^2^). Interestingly, there are solutions to the problem even for large place fields (Fig 2A, 2D and 2G). However, these solution are not robust. We quantified the robustness of the solution by lesioning different fractions of the grid cell inputs. Examples of the resulting firing rate maps (Fig 2A, 2D and 2G; bottom panels) indicate that the solution for the large place field is less robust to noise than the solutions for the other two place field sizes. A systematic exploration of the error rate (see Methods) reveals that the solution for the large size is highly sensitive to lesioning even a small fraction of grid cells (Fig 2H), even if the solution for small place field sizes is robust (Fig 2B).

Furthermore, the weights are unevenly distributed in the solution that produces the largest place fields (Fig 2I). Strong weights are only found to cells in the two modules with the largest grid spacings. The vast majority of inputs from grid cells, those with small spacings, have small weights on the output cell, raising the question of whether they are needed at all in the grid-to-place transformation and whether the large modules alone are sufficent. We therefore solved the classification problem on a grid cell population of equal size containing only cells in the two modules with the larger spacings. The classifier is able to find slightly more robust solutions for medium sized and large fields when the population has only large spacings. However, the solution remains sensitive to noise indicating that even those grid cells cannot trigger a robust place field of 35 cm radius.

We next investigated the reason why the place fields resulting from the simple grid-to-place transformation are unrealistically small. First, consider the case of competitive learning. It leads to a vector quantization of the input space (see for example [66, chapter 5]), which means that learning results in weight vectors that are similar to some input PV **w**_*i*_ = **p**(**r**_*i*_). Together with Eq (2), the cell activations are given by *h*_*i*_(**r**) = **p**(**r**_*i*_)^*T*^ **p**(**r**), which is the autocorrelation function of the PV. The autocorrelation is highest at zero offset and drops off very quickly, but, crucially, it rises again at periodic distances (see later in Fig 4C, purple line). To produce a cell that is active in a single place field around **r**_*i*_ from such an activation function, one only has to set a sufficiently high threshold *c*. The lower the threshold is, the larger the field. However, if *c* is too small, firing will occur outside the place field due to the periodicity of the activation map. The lowest threshold *c* that produces just one field, creates a field with a size of merely around 314cm^2^ in our model. Thus, competitive learning cannot produce single large fields. More generally, the periodic autocorrelation structure of the grid cell PV is problematic for any learning algorithm that one might use to find the weight vector **w**_*i*_ that produces a place field with a certain radius, say *R* = 30cm (∼ 2800cm^2^) at **r**_*i*_. The weight vector has to point into the direction of the PV **p**(**r**_*i*_) as well as into the directions of all the PVs at the locations that are within 30cm distance. At the same time, the weight vector has to point away from the directions of all the PVs outside the field. The challenge here is that the PV at **r**_*i*_ is nearly orthogonal to the PV at the locations **r** between 20cm and 28cm away (Fig 4C, purple line), but correlated with the PV further away that are outside the field, e.g., at about 45cm. While such weight vectors can be found (Fig 2D and 2G), these constraints make the solutions highly sensitive to noise (Fig 2E and 2H).

**Fig 3:**
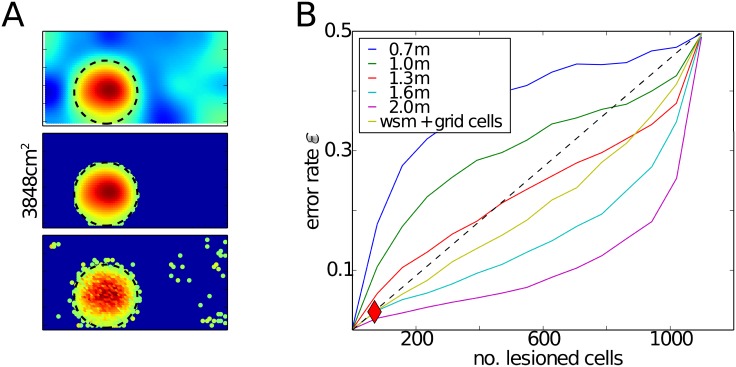
Solution of the classifier when grid cells have large spacings or when weakly spatially modulated cells are added to the input. **A**: Activation maps as in Fig 2 when the classifier is applied to a mixture of inputs from grid cells and weakly spatially modulated cells. **B**: Error rate as in Fig 2 when the classifier is applied to grid cell populations with a single large spacing (indicated by colored lines) or when weakly spatially modulated (wsm) cells are added to the grid cells with mixed spacings.

A simple solution to obtain robust and large place fields would be to increase the grid spacings in the input such that the second peak of the autocorrelation function appears outside the environment. To estimate the grid spacing required for this to occur, we performed simulations, in which all grid cells had the same large spacing, but different offsets. The orientations varied only to a negligible extent (std. 3 degrees). Our results reveal that grid spacing of at least 1.6 m would be required to generate a robust place field with a 35 cm radius. (Fig 3B). While such large spacing have been observed at the most ventral locations in MEC [67], the topographic projections from EC to the hippocampus [14] make it less likely that place cells in the most dorsal regions receive many inputs from these grid cells. Even more problematic for this simple solution is that the fact that multiple, spatially periodic place fields would appear if the walls of the environment were removed and the animal was allowed to explore the regions where the second, or further, peaks of the autocorrelation function occur.

In conclusion, our results suggest that it is rather unlikely that a linear transformation can produce place fields with realistic sizes based solely on grid cell inputs. Additional network mechanisms appear to be required to account for experimentally observed place field sizes.

### Mixing grid cells and weakly spatially modulated cells

A possibility to generate larger place fields is to add another input besides grid cells in the feedforward network. This input could be provided by weakly spatially modulated cells, which are abundant in the LEC [43–45] as well as in the MEC [38]. Adding inputs from weakly spatially modulated cells to the network, which already includes grid cells with multiple spacings, allows the classifier to find a robust solution for a place field with 35 cm radius (Fig 3A and yellow line Fig 3B). Since multiple modules alone are not sufficient to generate large place fields robustly (Fig 2H, blue line) and the addition of weakly modulated cells (Fig 3B, yellow line) make robust large place fields possible, we conclude that the robustness of large fields is caused by the weakly modulated cells and not the presence of multiple grid spacings.

**Fig 4:**
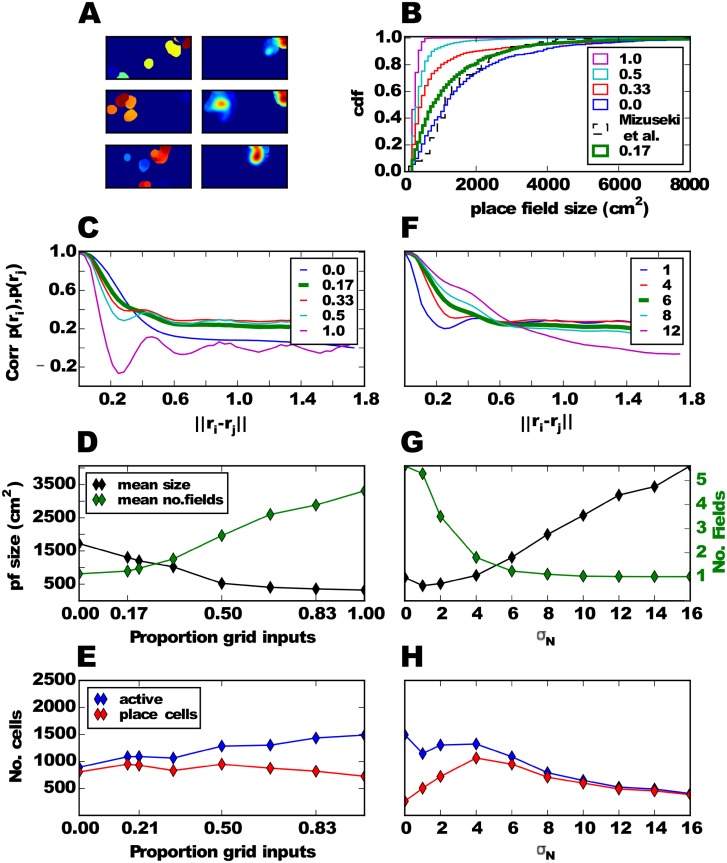
Generating place cells in a feedforward neural network model. **A**: Three examples of CA3 cell firing rate maps, one in each row, during learning (left column) and after learning (right column). **B**: Distributions of place field sizes in the CA3 population for different proportions of grid cells in the EC input. Thick green line shows the simulation with the default parameter. Dashed black line shows distribution for the rat CA3 [34]. **C**: Mean correlation of two input PVs as a function of the distance between their locations in space for different proportions of grid cells in the EC input. **D**: Mean place field size and mean number of fields per CA3 cell. **E**: Number of active cells and number of place cells. **F-H**: Similar to C-E, but varying the width *σ*_*N*_ of the smoothing kernel instead of the proportion of grid cells in the EC input.

We next asked whether a more biologically plausible feedforward model can learn to generate a realistic place cell population in a self-organized way (see Methods). To reproduce the results of previous models, we first investigated the feedforward neural network model with only grid cells in the EC inputs (fraction grid cells = 1.0). As expected the resulting place field sizes fell well short of the experimentally observed ones (Fig 4B, purple vs. dashed black line).

Our model can learn to transform mixed EC inputs into place cells with realistic field sizes (Fig 4). After a brief learning period, during which cells have several small fields, cells typically exhibit a small number of larger fields (e.g., Fig 4A). As the fraction of weakly spatially modulated cells increases, the size of resulting CA3 place fields increases (Fig 4B and 4D) and the number of fields per cell decreases (Fig 4D, green line). The size distribution and number of fields in the model matches the experimental results [34] best, when there is only a small fraction (< 17%) of grid cells among the EC inputs. Place fields are realistically sized, because the autocorrelation of the input PVs are single-peaked and wide (Fig 4C). For higher fractions of grid cells, a second maximum appears in the autocorrelation, thus forcing a higher threshold, which in turn leads to smaller place fields. A fraction of ≈ 17% grid cells in the EC inputs is consistent with data from the rat. Roughly half of EC consists of MEC and about one third of MEC cells projecting to the hippocampus are grid cells [38]. So, grid cells account for about 1/6 of EC cells in the rat.

In our simulations, not all CA3 become active in the environment, only around one third of CA3 cells do and almost all the active cells are place cells (Fig 4E). Curiously, the fraction of one third matches experimental findings [68–71] quite well.

Given the relative importance of weakly spatially modulated cells in generating realistic place field sizes, we further investigate the dependence on their properties, in particular the width of the smoothing kernel *σ*_*N*_ (see Methods). For the following analysis, we fix the proportion of grid cells at 1/6. If the kernel is narrow, the rate maps appear salt-and-pepper-like (Fig 1D) and the spatial autocorrelation is therefore rather narrow and dominated by the grid inputs (Fig 4F, blue and red lines). As a result, the field sizes are small, the mean number of fields per cell is significantly larger than one (Fig 4G) and very few of the active cells are place cells (Fig 4H). On the other hand, the autocorrelation of PVs is wide for larger kernel width. Consequently, the mean size of hippocampal place fields is larger in these simulations, there are fewer fields per cell (Fig 4G) and almost all active cells are place cells (Fig 4H). We choose an intermediate value of *σ*_*N*_ = 6 cm as default, since it also roughly matches the spatial information measured in LEC cells [44], but note that the exact procedure for generating the weakly modulated cells is not important for the model to replicate realistic place field sizes, only the spatial autocorrelation of PVs matters. In the following section all simulations are performed with this default value and with 1/6 of the input being grid cells and the rest being weakly spatially modulated cells.

### Robustness and stability of place cell responses

**Fig 5:**
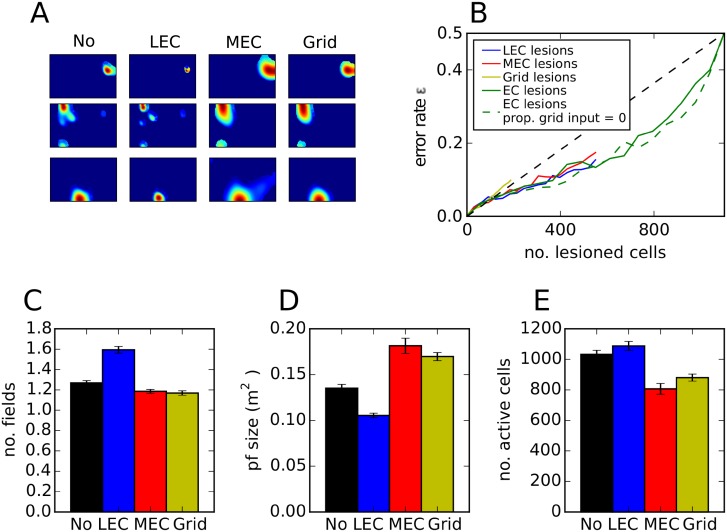
Effect of lesioning different EC inputs. **A**: Examples of the firing rate map of three CA3 cells, one per row. Columns show firing maps when no lesion is applied, the entire LEC is lesioned, the entire MEC is lesioned and when all grid cells are lesioned. **B**: Error rate as a function of the number of lesioned cells. Note that the network consists of of 550 LEC cells and 550 MEC cells (including 183 grid cells). **C-E**: Mean number of fields (C), place field size (D) and number of active cells when different EC inputs are lesioned. Standard error over ten simulations is indicated by error bars.

Since robustness is an important property of information processing in the brain, we next examine the model’s sensitivity to lesioning different types of EC inputs after the transformation has been learned. To facilitate the comparison with experimental results, we divide the EC input equally into MEC and LEC inputs. Grid cells make up one third of MEC cells. In this model, place fields appear largely preserved when all MEC, all grid cells or all LEC cells are lesioned (Fig 5A), suggesting that a subset of EC inputs are sufficient to maintain spatial selectivity in CA3. A systematic study, in which different fractions of input cells are lesioned selectively, reveals that place cells in our model are robust to lesions of LEC and MEC, but is sensitive to specific lesions of grid cells (Fig 5B). By adding grid cells, the model becomes more sensitive to noise than a model that receives only weakly spatially modulated cells (green dashed line in Fig 5B), confirming the analysis in Fig 2. Experimental studies indicate that, in MEC-lesioned rats, hippocampal place cell responses continue to be spatially selective in familiar environments, although their fields are broader and fewer cells are active [72, 73]. We therefore study place field properties after EC lesions in our model. If the entire MEC is lesioned, CA3 rate maps continue to be similar to those when the MEC input is present (Fig 5A), but the number of fields decreases slightly (Fig 5C), field sizes are larger (Fig 5D) and the number of active cells is smaller (Fig 5E). These modelling results are in good qualitative agreement with the experimental observations. If grid cells in MEC are selectively lesioned in our model, very similar effects are observed, suggesting that the experimental effect might be specifically due to the absence of grid cell firing. Complete lesions of LEC lead to contrary effects. The number of fields increases and the size decreases, which can be explained by the resulting higher proportion of grid cells in the input. Lu and Leutgeb et. al. (2013) [74] did not find changes in place field size in LEC lesioned rats, however the lesions consisted only of around 40% of the LEC and thus, were not complete (see Disscusion). To conclude, our model creates place cells that have realistic place field sizes, are robust and change their fields similarly as observed in lesion studies.

**Fig 6:**
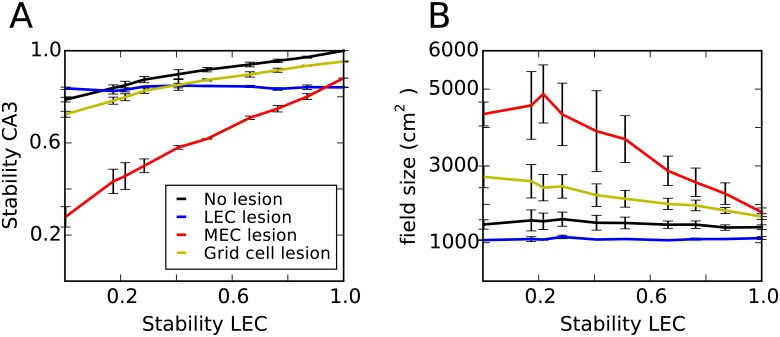
Stability of CA3 cells. **A**: Stability of CA3 firing maps between two visits of the same environment as a function of the stability in the LEC firing map. Lines of different colour show the result of lesioning different entorhinal inputs before the animal encounters the environment the second time. Errorbars show standard errors across three simulations. **B**: Mean field size in CA3 as a function of LEC stability.

Next, we tested the stability of CA3 place fields. Hippocampal place cells and cells in the MEC appear to have stable spatial firing maps during one recording session and between session in the same environment [4, 34, 44, 45]. By contrast, spatial firing is significantly less stable in LEC neurons especially in object-poor environments. Since LEC stability seems to depend on the properties of the environment, we parametrized the level of LEC stability (see Methods). We find that the model produces stable place fields with a constant field size for all stability levels (Fig 6, black line). Furthermore, lesioning the MEC leads to lower hippocampal stability and larger place fields. These effects are more pronounced when LEC stability is low. Lesioning the LEC had only minor effects. Thus, the pattern of stability in the model is in good agreement with experimental findings obtained in object poor environments [72, 74]. Moreover, our model predicts that the effects of MEC lesions on hippocampal place field size and stability are reduced in object rich environments, when LEC activity is more stable.

### Generating realistic place field sizes in a recurrent CA3 network

**Fig 7:**
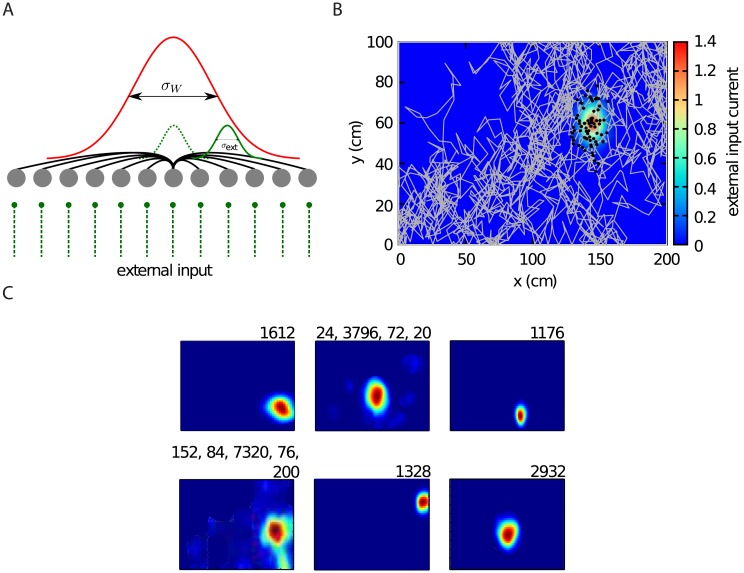
Recurrent connectivity enlarges the firing field of a place cell. **A**: A one dimensional representation of the CA3 network. Each cell receives a spatially selective input current (Eq 7) which is shown by green vertical bars. The extent of this input is indicated for two example cells by green bell-shaped curves (dashed and solid line). Because of the recurrent connectivity between the cells (red curve), the cell with a place field in the center can be activated even when the animal is located outside the space from where it receives spatial inputs, e.g., at the location of the solid green bell-shaped curves. **B**: Spatially modulated spiking of a representative CA3 cell (black dots) as the virtual animal randomly explored the environment. The external input current which determines the location of the place field center (Eq 7) has a Gaussian profile, indicated by the colourmap. Because of the recurrent excitatory connections, the place field of the cell is larger than the extent of the external input. **C**: Firing rate map of six place cells. The sizes of the detected place fields are indicated in the top right corner of each panel (in cm^2^).

We explore whether it is possible to generate realistic place field sizes based on grid cell inputs alone in a different network architecture. The idea is as follows. Each place cell receives narrowly tuned external inputs from grid cells, e.g., the cell depicted in the center of (Fig 7A, dashed green bell-shaped curve). This Gaussian input current is an abstraction of a transformation from a population of grid cells. A possible mechanism for the generation of this current is presented in the previous section. As the animal moves away from the center of the figure towards the right, the external input shifts to other cells (solid green bell-shaped curve). However, the cell in the center now receives excitatory drive from its recurrent connections (Eq 9), which receive external inputs. Therefore, the cells in the center continue to fire spikes even though they are no longer driven by external input, thus broadening their spatial firing field beyond the extent of the spatially modulated input (Fig 7B). Our simulations confirm that this mechanism generates place fields that are mostly centered around the external input and that closely match those observed experimentally (Fig 7C). We have verified previously that such a network maintains localized bumps of activity in the absence of spatially selective external inputs [62]. The presence of external inputs to CA3 in the current model forces the bump to form mostly at the location of the external input. However, some cells occasionally exhibit multiple place fields, which are the result of local instabilities. Since the place field centers are distributed randomly in the environment, there are small heterogeneities in the connection weight matrix Eq 9. As a result, the number and the strength of connections between excitatory cells in some regions in the network can be abnormally high. The excitatory synaptic inputs to the cells in these “hotspots” is higher than at other locations in the network. Besides, each cell receives a biasing input current, *I*_bias_ (Eq 6). As a consequence, the activity bump in the network does not always follow the external input that reflects the current location of the animal and occasionally cells outside the bump of activity become active, too. In other words, the bump trajectory will sometimes jump to parts of the network that do not correctly represent the animal’s location.

**Fig 8:**
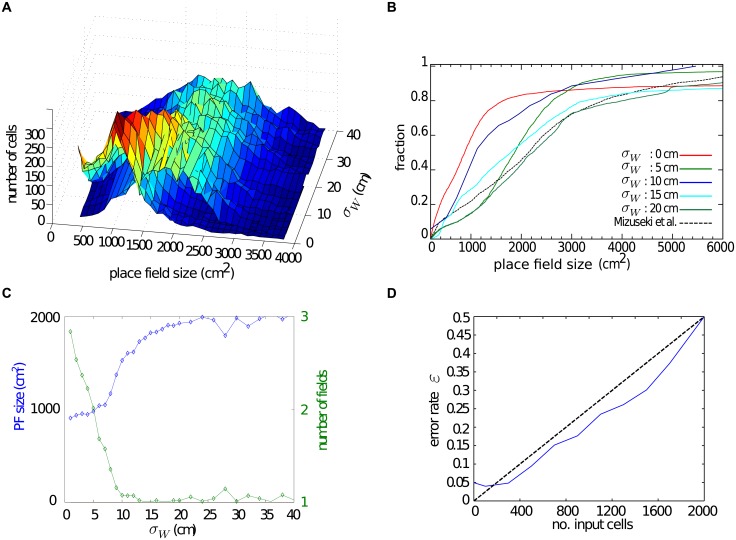
Properties of place cells generated by recurrent CA3 dynamics. **A**: The distribution of field sizes in the network for different connectivity kernel widths. Place fields are larger for wider connectivity kernels. **B**: Cumulative distributions of field sizes for different kernel widths. The distributions are similar to experimental results for a range of kernel widths. **C**: The median place field size (blue line) increases as a function of the kernel width. The average number of place fields of the cell in the network (green line) decreases with the kernel width. **D**: Fraction of bins with erroneous activity as a function of the number of cells without spatial input.

To investigate the effect of the recurrent dynamics systematically, we study the distribution of place field sizes as a function of the width of the connectivity kernel, *σ*_W_ (Fig 8A). The case *σ*_W_ = 0 is equivalent to the feedforward network. In this case the size of place fields is determined solely by the width of the external input *σ*_ext_. If the external input consists of grid cells only, the width is limited due to the structure of the spatial autocorrelation in grid cell PVs (Fig 4c, purple line). We therefore use a small value, *σ*_ext_ = 4c*m*. As expected, place fields are larger for wider connectivity kernels. To see this effect more clearly and to compare our results to the experimental data, we plot the cumulative fractions of the place field sizes for networks with different *σ*_W_ (Fig 8B). Increasing the kernel width up to ∼20 cm, makes place field larger (Fig 8C). Increasing the kernel width further does not have a strong effect on place field sizes, but makes it more difficult to generate stable place fields at all. This latter effect is due to an over-excitation in the system which results in multiple random active regions, which do not resemble well-defined place fields. For a wide range of connectivity kernel widths, the resulting distributions of place field sizes closely match the empirical data [34].

Other properties of place cells are reproduced by the recurrent network as well. For instance, cells have 1–2 place fields in nearly all simulations (Fig 8C) in agreement with experimental observations. The aforementioned network heterogeneity occasionally causes the cells to become active in multiple locations. By increasing the connectivity kernel width, the local structure of the network overcomes the effect of noise in the connectivity so that the average number of place fields decreases.

Like for the feedforward networks, we also study the robustness of the place fields in the recurrent network. We find that the network is robust to noise in the sense that when a randomly selected subset of inputs are removed, the error rate grows sub-linearly (Fig 8D). The error rate is nonzero even when the external input is intact because firing fields < 200 cm^2^ are not considered place fields and so are regarded as out-of-place-field firing when calculating the error rate (see Eq 11).

Based on our results, recurrent connectivity between CA3 place cells driven by grid cell inputs can account for the experimentally observed place field sizes. The spread of the recurrent connectivity is the determining element in the size and the stability of the resulting place fields.

## Discussion

We revealed that it is unlikely that robust place fields with realistic sizes are generated in a feedforward network driven by grid cell inputs alone [75], because of its structured spatial autocorrelation. This spatial structure is enhanced when grid cells express a common orientation [18] and experiments indeed show that grid cell orientations are clustered in rats [10, 46]. On the other hand, the grid symmetry reduces with increasing variation in the peak firing rates of the firing fields of individual grid cells [17, 75] and this variation has been found in rat MEC [2]. In this study, we adopt the conservative assumption that grid cell orientations are only clustered module-wise and different modules have different mean orientations distributed along the entire 0 to 60 degrees. Moreover, we introduce some variation between peak rates of individual firing fields, too. However, despite these symmetry breaking aspects, our general feedforward model shows that transformations from grid cells to large place fields are far from being robust. Although we have not mathematically proven that a robust transformation is impossible, our computational results leave only a narrow space for such a transformation to exist. It is thus likely that additional mechanisms have to come into play.

In some previous feedforward models, nonspatial input was present in addition to the grid cell input [21]. However, these had only a small effect on the place field sizes and could not generate realistic field sizes. [76] modelled the increase of hippocampal place field sizes along the dorsal to ventral axis by increasing the spacings of grid inputs along this axis and also by increasing the amount of nonspatial inputs at ventral locations. In this way, realistic place fields sizes could be generated in the ventral hippocampus, but not in dorsal hippocampus [76, Fig 5A]. Thus, both studies indicate that adding nonspatial input alone cannot account for realistic place field sizes.

Note that the problem is not that feedforward networks cannot generate large place fields at all, in fact, the abstract model we studied here does just that and it has been shown that a three layered network can learn any continuous mapping (universal approximation theorem) [77]. The problem is that there has to be a plausible neural mechanism for learning the transformation and the transformation has to be robust to noise and partial lesions of the network. One approach suggested that a place cell is the result of a Fourier transformation where grid cells with a common spatial phase are the basis functions [18]. To produce large place fields, the model relies on grid cells with very large grid spacings. One prediction of the model is that lesioning grid cells with large spacings leads to contraction of place fields, whereas lesions of cells with small spacings lead to an expansion of fields. A recent study tested this prediction experimentally by inactivating grid cells at three different locations along the dorsoventral axis of MEC, along which grid spacings increase systematically [78]. In contrast to the model predictions, inactivations at all MEC locations result in an expansion of place field size and a decrease in the number of place fields. Contradicting interpretations of that data exist, which defend the Fourier transformation model [78, 79]. However, both experimental findings are predicted by our feedforward model that includes inputs from weakly spatially-modulated cells.

Place cells have also been modelled as driven by cells carrying direct sensory information [80–82] or by cells with diffuse spatial information and head direction [83]. Our feedforward model moves beyond these studies by showing that place cells can be generated by a mixture of diffuse inputs and grid cells, that these cells have realistic and robust place fields, that they are temporally stable in conditions when large parts of the input are not, and by predicting their behaviour after entorhinal lesions.

The boundary vector cell (BVC) model suggests that place cell firing arise through the input from border cells in the MEC [84] in a feedforward network. The model can reproduce the empirical observation that the firing locations of place fields tend to maintain fixed distances to one or more boundaries following changes to the geometry of a familiar environment [9]. In principle, the model could produce realistic place field sizes, since border cells do not have a repetitive structure in their PV autocorrelation as grid cells do. Although, to our knowledge, this has not been shown explicitly. Here we use a more abstract cell class, the weakly spatially modulated cells, which do not systematically express firing fields at borders. Future work might examine whether adding BVC to MEC inputs can reproduce both realistic field sizes and appropriate responses when environmental borders are manipulated simultaneously.

### Limitations and future directions

One question that we did not address specifically in our computational study is how place fields with realistic sizes emerge in CA1. Experimental results indicate that spatial responses in CA1 are also driven by redundant inputs since CA1 place cells persist after inputs from either CA3 [85, 86] or layer III of MEC [73, 87] were removed. Our CA3 model of the feedforward network driven by grid cells and weakly spatially modulated EC cells can be applied to CA1 in a straightforward manner. The EC-CA1 pathway could account for preserved CA1 place cell firing after disconnection of CA3. CA1 could also inherit its spatial selectivity from CA3 via plastic feedforward connections [88, 89], thus accounting for preserved place cells in CA1 after lesions of MEC layer III. Mehta et. al. (2000) [88] suggested that spike-timing dependent plasticity at the CA3-CA1 synapses leads to larger place fields in CA1 as compared to CA3 cells and to the predictive shift of CA1 place fields with experience.

The current model of dorsal CA3 does not take into account the topography in the hippocampal formation, nor its anatomical structure. However, the grid spacing is organized topographically in the MEC [90]. Grid cells in the dorsal region of the MEC have small spacings and the spacing increases towards the ventral part. There is also topography in the connectivity pattern between EC and the CA regions of the hippocampus [47]. Whether this can account for larger place fields in ventral place cells [16, 18, 91] or whether a gradient of non-spatial input along the dorsal ventral axis is necessary [76] needs to be determined in our model. Moreover, there is a mixture of MEC and LEC inputs to the CA1 place cells along the septo-temporal axes [48]. Along the transverse axis there is a gradient of EC and CA3 inputs to CA1 [48, 92] as well as differences in place field characteristics [93]. While some studies hint at the importance of accounting for anatomical features of the hippocampal formation [94, 95], it remains unclear what their functional relevance might be for generating place-specific responses.

In the models we studied here, we focused on the spatial correlates of the hippocampal neurons’ spiking. Since in place cells, spatial responses and the timing of spikes are related through theta phase precession, it will be important to extent the model to account for temporal features of place cell firing. We have previously suggested that this temporal structure is perhaps the most important aspect of hippocampal activity [96, 97]. In a recent experiment, [98] observed that the spatial selectivity of place cells is abolished when the animal runs in a 2-D virtual reality environment, even though its visual appearance were matched to a real-world environment. The theta phase precession of their spiking activity, however, was not affected by the virtual reality.

### Redundant drivers of place cell activity

Place cells are surprisingly robust to MEC layer 3 [73], complete bilateral MEC [72], and partial LEC [74] lesions. In addition, place cells are robust to degradation of grid cells [25–28]. While some of these results could be accounted for by the robustness of the feedforward grids-to-places transformation [31], here we found that this model alone probably does not suffice to account for the observed sizes of place fields.

We suggest here that the robustness of place cells to lesions might stem from the fact that two redundant mechanisms exist for generating realistic place field sizes in CA3 cells: one relying on recurrent dynamics, and another one relying on weakly spatially-modulated cells. Both approaches reproduce hippocampal firing characteristics such as place field size, fractions of active cells, and average number of fields and they are robust to input perturbations. We hypothesize that the mechanisms are active in parallel and that this redundancy might account for the robustness of place cell activity to a number of invasive manipulations of the circuitry of the hippocampal formation.

Our model shows that although weakly spatially modulated EC cells have much lower spatial information than grid cells do, they still can drive the spatial selectivity of place cells. Spatial information might therefore not be the right measure to determine whether cells are driving hippocampal place cell firing. To generate large single place fields, the crucial requirement on the input are certain features in the spatial autocorrelation of its PVs. As long as nearby PVs are correlated in a sufficient large radius and the autocorrelation does not exhibit large values at larger distances, it can be transformed into place cells straightforwardly. Hence, a prediction of this model is that the PV of cells that project to place cells are of this kind, which can be verified experimentally.

In seeming contradiction to our conclusion that weakly spatially modulated cells are essential for generating realistic place fields in hippocampus, [74] reported that rats with a partial lesion of the LEC exhibit no differences in hippocampal field sizes in novel environments compared to controls. However, the mean lesion size in that study was around 40% of the LEC, which is equivalent to learning the transformation with a proportion of grid inputs of around $\frac{1/6}{1/2+0.6\times 1/2}\approx 0.21$ in our model. The model produces slightly smaller fields in this case (Fig 4C), but the difference is unlikely to reach statistical significance in the experiment. If, however, the LEC lesion were complete and CA3 recurrent connections were silenced, our feedforward model would predict significantly smaller place field sizes and more fields in CA3 (compare, for instance, proportion of grid inputs 1/6 to 1/3 in Fig 4C). While this would be a difficult experiment, it could be performed, in principle, with existing methods.

If, as our results suggest, grid cells are neither neccessary nor sufficient for the creation of place fields of realistic size in the hippocampus, what are they good for? A grid cell population can code for the animal’s location more accurately [99, 100] and more robustly [101] than a place cell population and hence they could provide additional information to the hippocampus. In line with this assumption, it has been suggested that grid cells are part of a path integration system in the MEC [16, 102] and they likely provide a spatial signal based on self-motion cues when other sensory inputs are not available, i.e., when other EC inputs are silent [103]. Indeed, LEC cells are receptive to sensory cues such as objects or odours [39–42] and their rate maps are less stable over time in object-poor environments as compared to grid cells [44, 45]. Thus, cells in the MEC, including grid cells, might be necessary for the formation of place cells in conditions where sensory inputs are very poor or absent. However, in other conditions our model shows, that weakly spatially modulated cells in the LEC alone are sufficient to generate realistic place cells. This reasoning could account for another set of recent recording experiments performed in novel environments. If the medial septum is inactivated, grid cells lose their spatially periodic activity pattern [25, 26]. If this occurs in a familiar or a small novel environment, place fields seem to be intact and stable [104, 105]. However, in a large novel environment, medial septum inactivation abolished CA1 firing fields and prevents the emergence of spatial stable firing [105]. To us these results suggest the following interpretation. In a familiar environment, inputs from weakly spatially modulated cells alone can maintain established place cell responses. The same inputs can generate a new spatial representation in a small novel environment because there is a sufficient number of distinct sensory features to uniquely identify a location. This is less likely, however, in a large novel environment, so that the grid cell input is needed to represent the spatial location by providing ideocentric information.

In conclusion, we have identified a new challenge in the transformation from grid cells to place cells, the place field size, and suggested two different mechanisms that can generate place fields with realistic sizes. Both mechanisms might be active in parallel and might be dissociable during different behavioural stages and through experimental manipulation.
